# Low Resource Complexity R-peak Detection Based on Triangle Template Matching and Moving Average Filter

**DOI:** 10.3390/s19183997

**Published:** 2019-09-16

**Authors:** Tam Nguyen, Xiaoli Qin, Anh Dinh, Francis Bui

**Affiliations:** 1 Biomedical Engineering Division, University of Saskatchewan, Saskatoon, SK S7N 5A9, Canada; minhtam_travinh@yahoo.com (T.N.); anh.dinh@usask.ca (A.D.); 2 Department of Electrical and Computer Engineering, University of Saskatchewan, Saskatoon, SK S7N 5A9, Canada; xiaoli.qin@usask.ca

**Keywords:** electrocardiogram (ECG), R-peak detection, triangle template matching, moving average filter, low resource complexity

## Abstract

A novel R-peak detection algorithm suitable for wearable electrocardiogram (ECG) devices is proposed with four objectives: robustness to noise, low latency processing, low resource complexity, and automatic tuning of parameters. The approach is a two-pronged algorithm comprising (1) triangle template matching to accentuate the slope information of the R-peaks and (2) a single moving average filter to define a dynamic threshold for peak detection. The proposed algorithm was validated on eight ECG public databases. The obtained results not only presented good accuracy, but also low resource complexity, all of which show great potential for detection R-peaks in ECG signals collected from wearable devices.

## 1. Introduction

Electrocardiogram (ECG) signal is commonly used to diagnose heart disease. There are various studies and applications in this field. For example, in the work by the authors of [1], a method based on polygonal approximation was proposed to detect fiducial point of ECG QRS complex. This method has higher accuracy than other signal compression techniques, but requires more energy consumption. In the work by the authors of [2], the authors developed a single chip based wearable wireless ECG monitoring system. However, they did not implement further post-signal processing analysis. The automatic detection of R-peaks is considered a classic ECG signal processing problem and has been extensively investigated. Recently, there were several notable studies [3,4,5,6,7,8,9,10]. In the work by the authors of [3], the combination of wavelet transform, derivatives, Hilbert transform, and adaptive thresholding was proposed. This method can provide a high detection accuracy. Although this algorithm does not need to learn from previously detected R-peaks, it is complex and only validated on the MIT-BIH arrhythmia database, QT database and noise stress database. Two R-peak detection algorithms were proposed based on envelopment and K-mean clustering technique in the work by the authors of [4]. The first algorithm exhibits high level detection accuracy and is free of parameters, but can only be used off-line. A domino error effect can occur if the training process is not sufficient. In addition, most of these algorithms are only suitable for normal heartbeats. In the work by the authors of [5], the second-order Volterra filter and averaging filter were used to estimate the ECG envelope. One single threshold was used to define the interested block containing R-peaks. Only the MIT-BIH arrhythmia database was tested in this study. In addition, the computational complexity of this algorithm is high.

Recently researchers focused on developing efficient detection algorithms suitable for wearable ECG devices. In the work by the authors of [6], one algorithm was proposed to detect R-peaks of real ECG signals measured by a proposed prototype, but only few public ECG signal databases were evaluated. In the work by the authors of [7], a simple real-time R-peak detector with low computational cost was proposed. Similar to many previous works, the authors of [7] customized input parameters of the algorithm for optimal performance on the MIT-BIH arrhythmia database [8], which may not be amenable to other databases. Moreover, it is not robust to noise.

According to the comprehensive review by the authors of [9], Elgendi’s algorithm [10] was recently evaluated as the best algorithm to detect R-peaks in portable devices due to robustness to noise, parameter choice, and numerical efficiency. The Elgendi’s algorithm is based on a Butterworth band-pass filter, squaring, and two moving average filters. The optimal frequency band of the band-pass filter is 8 Hz to 20 Hz. After filtering, the signal is squared point by point to enhance large values and boost high-frequency components. Next, two moving average filters are used to generate blocks of interest that potentially contain R-peaks. The blocks of interest are found by comparing the differences between the outputs of two moving average filters in which the longer window is used to create a threshold. To increase the detection accuracy in a low signal-to-noise ratio ECG signal, the statistical mean of the input signal is used to generate an offset in the threshold. Finally, the location of the maximum of absolute values within each block of interest is considered as the R-peak. Although Elgendi’s algorithm is computationally efficient, it needs a global ECG record to calculate the threshold. This results in a latency dependent on the input signal length.

One well-known real-time R-peak detection algorithm was proposed by Pan-Tompkins [11]. This classical algorithm can process and display the detection result for every sample after a learning period. However, the complexity of the Pan-Tompkins algorithm is high and the detection accuracy is moderate compared with Elgendi’s algorithm. For example, as described in the work by the authors of [10], the algorithm of the authors of [11] performed much worse than the algorithm of the authors of [10] on databases IAFDB and INCARTDB. In the work by the authors of [12,13], a triangle shape matching filter was developed using three samples of each side to distinguish R-peaks from T waves and P waves. The basis of this idea is the fact that the slope of R-peak is sharper than slopes of T wave and P wave. To detect R-peak, the vertex angle of the triangle shape is also used. Since the angles of the triangle are not uniform in polarity and magnitude, this method is also not robust to noise. Furthermore, in the works by the authors of [14,15], the triangle shape matching was applied to estimate the location of the QRS complex to design the low-pass filter for electromyogram (EMG) removal. In these studies, the triangle shape matching filter was not used directly for accurate R-peaks detection, but the template shows a great potential to estimate a block which contains QRS complex. In addition, the advantage of triangle shape matching is simple and can be processed sample-by-sample.

In our work, the proposed algorithm is developed by combining triangle template matching [12,13,14,15] and moving average filter [10,16] for R-peaks detection, in order to address some shortcomings as mentioned above. Although triangle template matching can provide the location of the windows containing the QRS complex on a sample-by-sample basis, the moving average filter gives information on the R amplitude, which can be used for defining the dynamic threshold to detect R-peaks with high robustness to noise. The proposed work was evaluated with four objectives: robustness to noise, low latency processing, low resource complexity, and automatic tuning of parameters on eight public databases. Compared to other R-peak detection algorithms, the obtained results demonstrate that the method herein proposed exhibits improved performances with respect to all four objectives.

## 2. Materials and Methods

### 2.1. Training Data Set

The proposed R-peak detection algorithm was trained on the MIT-BIH arrhythmia database [8]. For each record of the database, independent cardiologists annotated all peaks. In this work, all non-beat annotations defined by Physionet are ignored. With this assumption, there are 109,494 annotated beats in this database. This number is consistent with the results of other studies [17,18,19,20,21,22,23,24,25]. To be consistent with other studies of R-peaks detection, quantitative comparisons in terms of sensitivity (S), positive predictivity (P), and detection error (DER), as defined in Equations (1)–(3), are studied.

(1) $$S(\%)=\frac{\mathrm{TP}}{\mathrm{TP}+\mathrm{FN}}$$

(2) $$P(\%)=\frac{\mathrm{TP}}{\mathrm{TP}+\mathrm{FP}}$$

(3) $$\mathrm{DER}(\%)=\frac{\mathrm{FN}+\mathrm{FP}}{\mathrm{TP}+\mathrm{FN}}$$

The true positive (TP) is defined as the number of QRS complexes detected as QRS complexes. False negative (FN) is the number of QRS complexes which have not been detected, and false positive (FP) is the number of non-QRS complexes detected as QRS complexes. The sensitivity represents the percentage of true beats that are correctly detected, whereas the percentage of detected true beats is presented by the positive predictivity. For the TP and FN calculations, the beat-by-beat comparison standard of the Association for the Advancement of Medical Instrumentation (AAMI) [26] is used.

### 2.2. Testing Data Set

Seven public databases were used for evaluating the proposed method: the Noise Stress Test database [27], the meta-data set QT Database [28], The Long-Term ST Database [29], the T-Wave Alternans Challenge Database [30], the MIT-BIH Supraventricular Arrhythmia Database [31], the MIT-BIH Normal Sinus Rhythm Database [32], and the MIT-BIH Atrial Fibrillation Database [33]. The sampling rate varied across the different databases; hence, a resampling procedure was applied with a common sampling rate of 360 Hz.

## 3. The Proposed R-Peak Detection Algorithm

**Figure 1:**
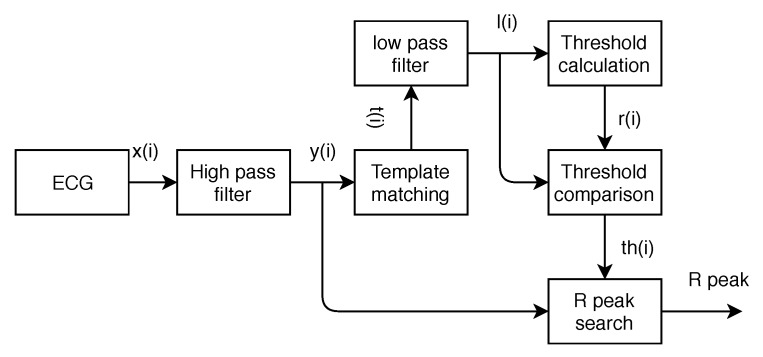
The proposed algorithm structure, consisting of six steps.

The diagram of the proposed algorithm is shown in Figure 1. The method consists of six steps: high-pass filter, template matching, low-pass filter, threshold calculation, threshold comparison, and R-peak search. The high-pass filter can reduce the interference of baseline wander and T-wave. Template matching is achieved by the multiplication of the slopes of two adjacent segments of a sample. This step will enhance the R-peak height and also boost the amplitude of high frequency signals. As the template matching is a nonlinear operator that often generates high frequency noise, a low-pass filter is applied to remove such noise in the third step. In the next step, an averaging filter is designed to create a dynamic threshold. In the fifth step, candidate blocks of R-peak are established. Finally, the peak locations will be sought in candidate blocks.

**Figure 2:**
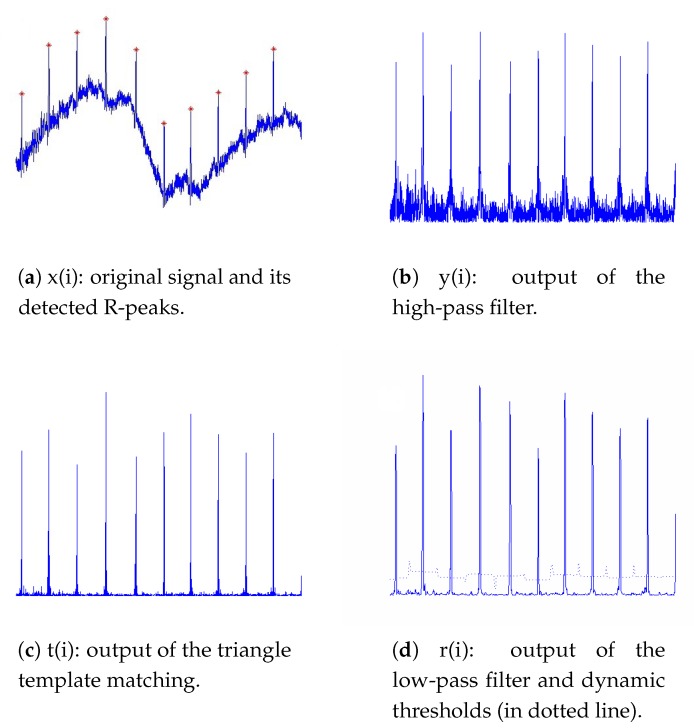
Signals at different steps in the algorithm.

Figure 2 shows the signals in time domain for each step in the proposed algorithm. Details of each module are described as follows.

### 3.1. High-Pass Filter

The purpose of the high-pass filter is to eliminate the baseline wander caused by respiration, muscle contraction, and electrode impedance changes related to perspiration or movement. In addition, it can also reduce the T-waves in ECG signals. The analysis in the work by the authors of [11] shows that the power of P and T waves and motion artifacts concentrates in frequencies lower than 5 Hz. Therefore, a cut-off frequency higher than 5 Hz is typically selected. In this work, a simple high-pass filter based on a moving average [10,16] is designed. The filter used has the following form.

(4) $$\hat{y}\left(i\right)=x\left(i\right)-\frac{1}{2N+1}\sum_{j=-N}^{N}x\left(i+j\right)$$

where *N* defines the observation window length. The output of this filter can be written as

(5) $$\hat{y}\left(i+1\right)=x\left(i+1\right)-\frac{1}{2N+1}\sum_{j=-N}^{N}x\left(i+j+1\right)$$

(6) $$\hat{y}\left(i+1\right)=\hat{y}\left(i\right)+x\left(i+1\right)-x\left(i\right)+\frac{1}{2N+1}\left(x\left(i-N\right)-x\left(i+N+1\right)\right)$$

The transfer function of the high-pass filter is expressed as

(7) $$H\left(z\right)=\frac{z-1+\frac{1}{2N+1}\left(z^{-N}-z^{N+1}\right)}{z-1}$$

In this filter, the cut-off frequency is specified by *N*. For a particular database or hardware device in which the sampling rate is known, the value of *N* can be determined by using the Algorithm 1 for window length selection. Since a low group delay is desirable, the first value of the window length (*N*) that satisfies the condition at line 10 in the Algorithm 1 is selected. For example, N=25 is selected to meet a cut-off frequency of 5 Hz for a signal with a sampling rate of 360Hz.

To detect the negative QRS complex in ECG signal, the output of the filter y(i) is defined as

(8) $$y\left(i\right)=|\hat{y}\left(i\right)|$$

**Algorithm 1:** Pseudo Code for the Window Length Selection Algorithm
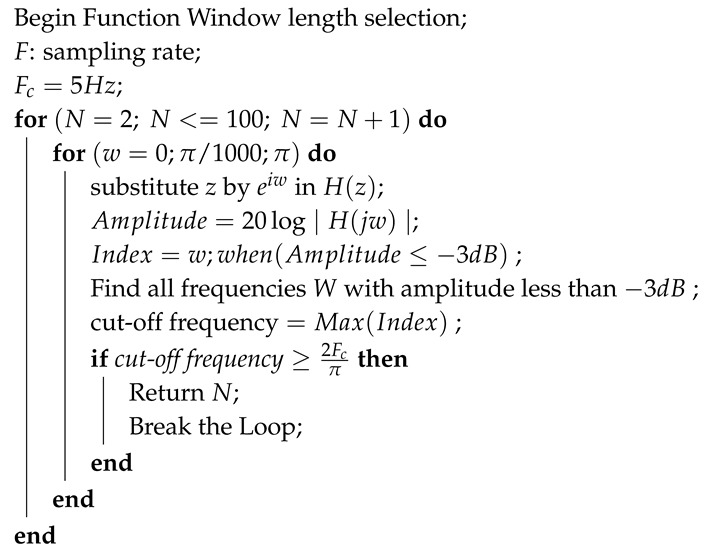



### 3.2. Triangle Template Matching

The goal of the triangle template matching is to rectify the differentiated ECG signals to amplify QRS complexes regardless of their polarity in the original input. Similar QRS detection techniques based on matched filters were studied in [34,35,36,37,38,39,40,41,42]. A QRS complex is created when the ventricles depolarize prior to their contraction [3]. In addition, the QRS complex has the largest amplitude and a sharp upward slope of any ECG signal. To accentuate the QRS complex, triangle template matching is defined as

(9) $$t\left(i\right)=\left(y(i)-y(i-s)\right)\left(y(i)-y(i+s)\right)$$

The output of Equation (9) determines the degree of matching between the triangular shapes of the ECG signal and the template. We assume that the shape of the QRS complex is nearly symmetric. To get a suitable R-peak enhancement for subsequent processing, the length of the window for the triangle template matching should be commensurate to the QRS complex width. Typically, the width of a QRS complex ranges from 80 ms to 120 ms. Accordingly, to achieve robustness, e.g., handling abnormal heart beats with large HRV, a 40 ms duration is chosen for the template window. With a particular sampling rate, the value of s in Equation (9) can be determined. For example, with a 360 Hz sampling rate, s=7 is typically selected. We retain the positive values of t(i) in Equation (9). High frequency noise (such as EMG) does not have a similar template to the QRS complex, so its response to the proposed matched filters should be smaller than that for the QRS complex. In other words, template matching also functions as a form of selective filter.

### 3.3. Low-Pass Filter

Ideally, the output of the triangle template matching should be locally maximized at R-peaks. Hence, the indices of the peaks of t(i) can be considered as potential R-peaks. However, t(i) also exhibits many noisy peaks. The reason is that the template matching is a nonlinear operator that may generate additional high frequency components. To reduce such noisy peaks, a subsequent low-pass filter is applied. In this work, a low-pass filter takes the form of

(10) $$l\left(i\right)=\frac{1}{2L+1}\sum_{j=-L}^{j=L}t(i+j)$$

The low-pass filter can also be written as

(11) $$l\left(i+1\right)=l\left(i\right)+\frac{1}{2L+1}\left(t\left(i+L+1\right)-t\left(i-L\right)\right)$$

So that the corresponding transfer function is expressed as

(12) $$H_{l}\left(z\right)=\frac{z^{L+1}-z^{-L}}{\left(2L+1)(z-1\right)}$$

The purpose of this low-pass filter is to retain the authentic potential R-peaks in t(i). In the work by the authors of [43], the authors observed that the band-pass filter with a center frequency of 17 Hz is optimal for detecting QRS complexes. Thus, we assumed the frequency of the possible R-peaks in t(i) is between 5 and 35 Hz. In our case, with a sampling rate of 360 Hz and a cut-off frequency of 35 Hz, L=5 is typically selected.

### 3.4. Threshold Calculation

The idea of threshold calculation is inspired by the Elgendi’s algorithm [10]. In this work, the signal l(i) in Equation (10) plays a similar role to emphasize locations of QRS complexes. The candidate blocks containing R-peaks are generated by comparing the l(i) with a dynamic threshold th(i):

(13) th(i)=βMA(i)+θ

where β and θ are defined coefficients, MA(i) is an averaging filter constructed as

(14) $$MA\left(i\right)=\frac{1}{2M+1}\sum_{j=-M}^{j=M}l\left(i+j\right)$$

**Figure 3:**
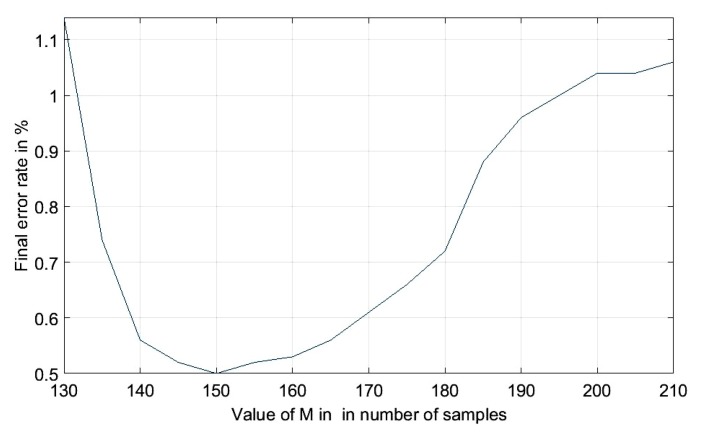
Influence of window length of the dynamic threshold on the overall error rate based on brute force optimization with fixed β=2.5 and θ=206.

The th(i) threshold in Equation (13) is similar to the first dynamic threshold value $THR_{1}$ in Elgendi’s algorithm. It is used to find the QRS in one heartbeat. Therefore, the window length (2M+1) of the averaging filter in Equation (14) should approximate the duration of a heartbeat, which is of 360 samples (for a sampling frequency of 360 Hz). However, in practice, the heartbeat duration varies. The result of a brute force exhaustive optimizer to find the optimum, *M*, using the training data set is presented in Figure 3. It can be seen from Figure 3 that when *M* equals to 150, the lowest error rate was achieved.

To enable a rapid R-peak search in candidate blocks, the interested block width should be small. Therefore, the coefficient β is inserted into Equation (13). When β is small, the width of the interested block is big. However the scale of β does not have significant effects on the final performance. The experimental results show that its value can vary between 2 to 4 in many databases. In our work, we fix β=2.5. The coefficient θ is also added in Equation (13) to reduce the number of false positive detection.

There is a trade-off in the choice of θ in which larger values are more suitable for R-peak detection in noisy ECG signals. Meanwhile, a reasonable value must be maintained to detect a R-peak with small amplitude. Experiments show that θ could be one-fourth of the statistical mean of the output of the low-pass filter. The drawback of this calculation is that this method does not guarantee low latency. To address this issue, we proposed an iterative Equation (15) to get a dynamic threshold in low latency. In our work, the statistical mean of l(i) for the “training database” is used for parameters initialization. For this database, this value is ~824, thus θ=206. Finally, the threshold th(i) is calculated as

(15) $$th\left(i+1\right)=th\left(i\right)+\frac{1}{2M+1}\left(l\left(i+M+1\right)-l\left(i-M\right)\right)+206$$

### 3.5. R-Peak Search

**Figure 4:**
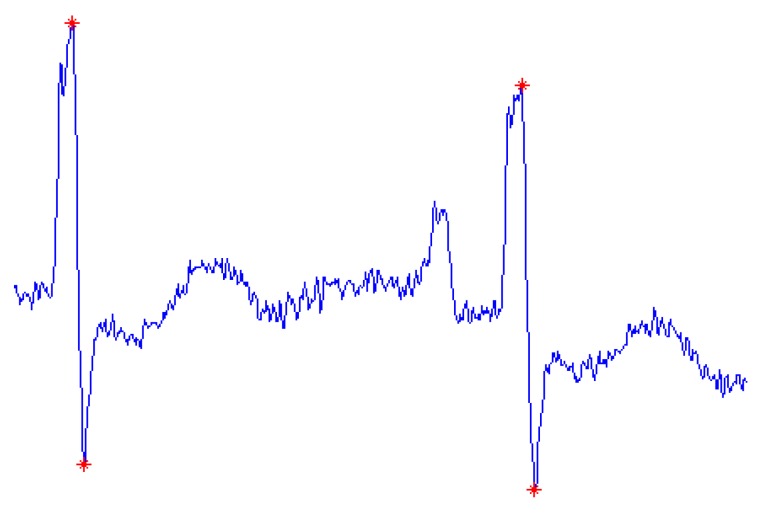
Multiple detected points in one QRS complex.

The actual R-peaks will be at the blocks of interest, where l(i) is higher than the threshold th(i). The index of the maximum point of the search window is considered as the index of an R-peak. The experiment shows that the possible maximum width of the search window is half of the normal QRS complex width. As the value of y(i) can also be large at Q or S peaks, there are multiple detected points within one QRS complex as illustrated in Figure 4. With the reasonable assumption that the maximum heart rate is ~206 bpm [44], the distance between two continuous R-peaks cannot be smaller than 272 ms, so an error correction step is applied whenever two detected R-peaks are too close to each other. The detected R-peak of larger amplitude is retained, while the other one is eliminated. The pseudocode of the proposed R-peak search method is shown in Algorithm 2.
**Algorithm 2:** Pseudo Code for the R-peak detection Function.
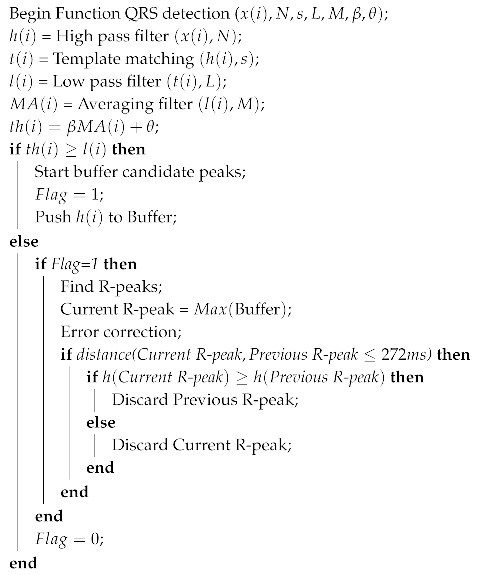



### 3.6. Computational Cost

The proposed algorithm was implemented in a desktop. The processing time of the algorithm is small compared with the input length duration. The comparison details will be presented in the processing time section. For readers potentially seeking to implement the algorithm on hardware devices, such as with FPGA technology, the computational resource costs are shown in Table 1. For comparison, the estimated computational resource costs of Elgendi’s algorithm and a recent novel template matching technique [41] are presented in Table 2 and Table 3.

Generally, the proposed algorithm exhibits lower computational resource cost than the recent matching technique and moving averaging filtering methods when counting the number of registers, adders, multipliers, or comparators used. This is because the triangle template matching and proposed iterative dynamic thresholding methods are applied. The QRS complexes are well rectified after using the triangle template matching, and the iterative dynamic threshold calculation is independent of the the window length. In contrast, Elgendi’s algorithm implemented averaging filtering twice and the calculation cost depends on the window length. Normally, the more operations of averaging filtering are taken, the higher computation resource cost will be.

### 3.7. Latency Cost

To initialize the proposed algorithm, input data is first buffered with 0.833 s (300 samples) to calculate the threshold in Equation (13). Then, the processed data is calculated sample-by-sample. In addition, the complexity of the algorithm is very small. Therefore, the total latency cost of the proposed method can be estimated as 0.833 s.

## 4. Results and Discussion

### 4.1. Selected Algorithms for Evaluating the Proposed Work

In this work, the performance of the proposed algorithm on many published databases is evaluated and compared against other two state-of-art methods: the Pan-Tompkins [11] and the Elgendi’s algorithm [10]. The Pan-Tompkins method represents the most well-known classical real-time R-peak detector, whereas the second algorithm was recently evaluated as the best algorithm to detect R-peaks in portable devices.

It is noted that not all authors of previous studies presented complete details on how comparisons of annotated beats with their detected beats were performed. In this work, the standard of AAMI [26] is applied. The use of this standard is consistent with the work by the authors of [25].

### 4.2. Evaluating on the MIT-BIH Arrhythmia Database

Table 4 presents the performance of the proposed algorithm with its default parameters against all records. The average DER value is 0.49%. The proposed algorithm performs well on most records and their maximum DER is below 1.6%, except on three records, 207, 203, and 210. The error of the record 207 occurs due to the significant false positive errors during the flutter episodes. Physionet has considered record 207 as an extremely difficult record. According to the latest version of the annotations published in June 2010 by Physionet, there are 1860 beat annotations. In our work, only five beats were not detected. There are 472 ventricular flutter peaks in this record and the ventricular flutter peaks are considered as non-beat annotations. The proposed algorithm detects a number of ventricular flutter peaks in this record. That is why there are significant false positive errors. However, some previous works, such as the work by the authors of [10], excluded episodes of ventricular flutter beats in their ground truth.

In record 210, the average heartbeat is about 240 samples. The corresponding optimal value of parameter *M* in Equation (14) should be 120. Since the default *M* value of 150 was used, the obtained performance was suboptimal. In record 203, sudden amplitude changes of some beat pulses led to misdetection in our work.

### 4.3. Evaluating with Other Public Data

For further evaluation, performance comparisons between the proposed method and the other two selected well-known methods for different databases were evaluated on seven public databases. The results are shown in Table 5. It can be seen from Table 5 that the proposed algorithm performs better in terms of DER. This indicates that the proposed algorithm also can work well with different typical clinical ECG signals.

### 4.4. Evaluating the Robustness to Noise of R-peak Detection Algorithms

In the MIT-BIH arrhythmia database, there are many records, such as 121, 202, 200, 217, 105, and 108, that are strongly affected by noise including baseline wander and muscle noise. These records were used to evaluate the robustness to noise in some previous works [5,25]. Table 6 shows comparisons of the DER values of the proposed method with the other nine studies. The biggest DER value in each column is in bold. It can be seen from Table 6 that the DER value of the proposed algorithm is comparable with previous works in the same records, which are contaminated heavily by noise.

### 4.5. Comparing Performance Between the Proposed and Elgendi’s Algorithms in Some Specific Signals

#### 4.5.1. Record 109 of the MIT-BIH Arrhythmia Database

**Figure 5:**
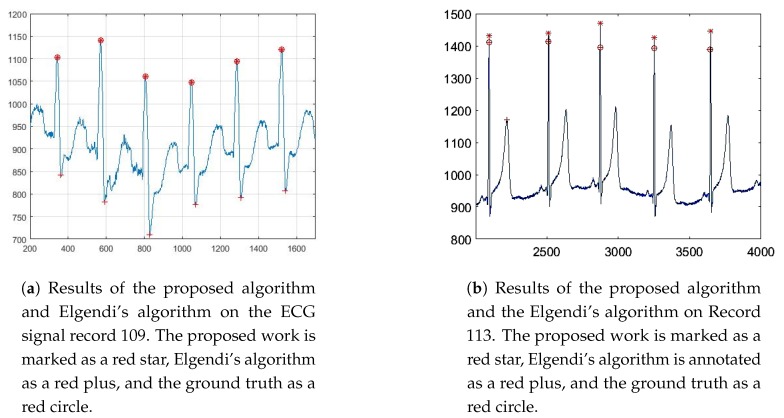
Results of the proposed algorithm and Elgendi’s algorithm on record 109 and 113.

Elgendi’s algorithm took a band-pass filter to concentrate QRS complexes with the cut-off band of 8–20 Hz. With this filter, the baseline wander and high frequencies are almost removed. However, previous studies showed that the power of QRS complex is concentrated in the band of 5 Hz–35 Hz. Thus, a small cut-off band can lead to the distortion of the sharpness of the R-peak. Consequently, some locations of R-peaks detected by Elgendi’s algorithm are not correct. It should be noted that this type of error is not reflected directly in those quantitative metrics due to the false detection is very close to the ground truth. Figure 5a shows the detected R-peaks of the proposed work and Elgendi’s algorithm for the signal of record 109 from sample number 200 to 2000.

#### 4.5.2. Record 113 of the MIT-BIH Arrhythmia Database

Record 113 does not have much noise, but P-peaks in this record have high amplitudes. These P-peaks result in outputs of the first moving average filter in Elgendi’s algorithm that may be bigger than the first threshold. Moreover, widths of these P-peaks are also larger, which means the second threshold of Elgendi’s algorithm does not help eliminate the false “block of interest”. The error rate of Elgendi’s method on record 113 is 5.52%, and most peaks are false positive detected. As the slope of the P-peaks is always smaller than the slope of the R-peak, the triangle template matching of our proposed method can amplify the difference between R-peak and P-peak. In addition, the “error correction” step also provides an advantage in canceling “wrong detected R-peaks”. Figure 5b shows the detected R-peaks by the two methods for the samples 2000 to 4000 from record 113.

### 4.6. Processing Time

In Table 2, the total computational resource cost of the proposed algorithm was presented. In this section, we compared the processing time of each method. The chosen methods were implemented in MATLAB version 2015b on a desktop of ${\mathrm{Intel}}^{TM}$i5-4570 CPU 3.2 GHz, 8 GB RAM. Table 7 shows the comparison results on average computational time over 10 trials in processing the MIT-BIH arrhythmia database.

As data shown in Table 7, the proposed method runs faster than the two state-of-art algorithms even with a better accuracy for a huge potential in real-time applications.

### 4.7. Parameters Setting

There are only six parameters in our work and their value can be tweaked according to different databases. The window size of the high-pass filter, parameter (N) in Equation (4); triangle template matching, parameter *s* in Equation (9); and low-pass filter parameter (L) in Equation (10) can be determined automatically when a sampling rate is known. The size of the moving average filter parameter (M) in Equation (15) used to define the threshold depends on the heart rate, and it is hard to be predicted. Our approach is to estimate it by using a normal heart rate. The coefficient β in Equation (13) is fixed to 2.5. In fact, the value of this parameter does not strongly affect final performance. The coefficient θ in Equation (13) depends on the amplitude of the R-peak, and it is also difficult to predict its value in an efficient way. The proposed method takes one-fourth of the statistic mean of the outputs from low-pass filter. In addition, we took the default value trained from the MIT-BIH database and tested on other seven databases, results showed that the default value is applicable for many databases with good performance.

## 5. Conclusions

The proposed method is based on a triangle template matching and moving average filter and aimed to tackle common challenges in the detection of R-peaks in ECG signals. The results show that the proposed algorithm performs better compared to previous works in trading off four objectives: robustness to noise, low latency processing, low resource complexity, and automatic tuning of parameters.

The previously published algorithms are mostly computationally complicated and require impractical assumptions, e.g., they typically need global statistical knowledge of the entire input signal. In contrast, the proposed algorithm not only exhibits low complexity, but also, even with default parameters, it is already highly competitive against the classical Pan-Tompkins’ and the well-praised Elgendi’s algorithm for many publicly available ECG databases. In addition, for additional performance gains, including optimization in a new or customized application scenario, the parameter tuning process is also straightforward, at the expense of a modest increase in latency complexity. Altogether, these desirable characteristics reinforce the practical feasibility of the proposed method for wearable mobile applications.

One disadvantage of the proposed work is that the value of *M* in Equation (15) is fixed. This value should be updated with the real heart rate to have the best performance. One possible alternative solution is to have a learning phase to estimate the real heart rate. However, noisy ECG signals often result in a poor estimation of the real heart rate during the learning phase. Given this challenge, the learning phase should be long enough to overcome it, but at the expense of an increase in latency. 

## Figures and Tables

**Table 1:** Computational resource cost of each step in the proposed algorithm.

Step	Eq.No.	Register	Adder	Multiplier	Comparator
High pass filter	6	1	4	1	
Triangle Match Filter	9	1	2	1	
Low pass filter	11	1	2	1	
Threshold Calculation	13	1	3	1	
Threshold Cmp, R-peak search					1
Total		4	11	4	1

**Table 2:** Computational resource cost of each step in Elgendi’s algorithm.

Step	Eq.No.	Register	Adder	Multiplier	Comparator
Band pass filter		1	7	8	
Squaring	1			1	
Averaging filter	2&3	2	6	2	
Interested block	4		2		1
Threshold		3			2
R-peak detection					1
Total		6	15	11	4

**Table 3:** Computational resource cost of each step in Hu’s algorithm [41].

Step	Eq.No.	Register	Adder	Multiplier	Comparator
Local normalization	1	1		1	2
Straight line fitting	2		62	32	
Angle calculation	4	2	3		
R-peak detection		1	2		2
Total		4	67	33	4

**Table 4:** Performance Evaluation of the proposed algorithm for the MIT-BIH Arrhythmia Database.

Record	Total	TP	FN	FP	S(%)	P(%)	DER(%)
100	2273	2273	0	0	100.00	100.00	0.00
101	1865	1864	1	4	99.95	99.79	0.27
102	2187	2186	1	0	99.95	100.00	0.05
103	2084	2084	0	0	100.00	100.00	0.00
104	2229	2227	2	13	99.91	99.42	0.67
105	2572	2563	9	22	99.65	99.15	1.21
106	2027	2027	0	3	100.00	99.85	0.15
107	2137	2136	1	0	99.95	100.00	0.05
108	1763	1750	13	14	99.26	99.21	1.53
109	2532	2530	2	1	99.92	99.96	0.12
111	2124	2123	1	1	99.95	99.95	0.09
112	2539	2539	0	1	100.00	99.96	0.04
113	1795	1795	0	0	100.00	100.00	0.00
114	1879	1875	4	5	99.79	99.73	0.48
115	1953	1953	0	0	100.00	100.00	0.00
116	2412	2396	16	2	99.34	99.92	0.75
117	1535	1535	0	0	100.00	100.00	0.00
118	2278	2278	0	1	100.00	99.73	0.48
119	1987	1987	0	1	100.00	99.95	0.05
121	1863	1861	2	1	99.89	99.95	0.16
122	2476	2475	1	1	99.96	99.96	0.08
123	1518	1518	0	0	100.00	100.00	0.00
124	1619	1619	0	2	100.00	99.98	0.12
200	2601	2600	1	6	99.96	99.77	0.27
201	1963	1954	9	0	99.54	100.00	0.46
202	2136	2134	2	0	99.91	100.00	0.09
203	2980	2924	56	28	98.12	98.12	2.82
205	2656	2650	6	0	99.77	100.00	0.23
207	1862	1857	5	172	99.73	91.51	9.52
208	2955	2944	11	2	99.63	99.63	0.44
209	3005	3005	0	0	100.00	100.00	0.00
210	2651	2581	70	1	97.36	99.96	2.68
212	2748	2748	0	0	100.00	100.00	0.00
213	3251	3251	0	0	100.00	100.00	0.00
214	2263	2261	2	1	99.91	99.96	0.13
215	3365	3363	2	0	99.94	100.00	0.06
217	2208	2208	0	0	100.00	100.00	0.00
219	2158	2154	0	1	100.00	99.95	0.05
220	2048	2048	0	0	100.00	100.00	0.00
221	2427	2427	0	1	100.00	99.96	0.04
222	2483	2480	3	2	99.88	99.92	0.20
223	2605	2605	0	1	100.00	99.96	0.04
228	2056	2054	2	19	99.90	99.08	1.02
230	2256	2256	0	0	100.00	100.00	0.00
231	1571	1571	0	0	100.00	98.99	1.02
232	1780	1780	0	5	100.00	99.72	0.28
233	3079	3079	0	0	100.00	100.00	0.00
234	2753	2753	0	0	100.00	100.00	0.00
Total	109494	109270	224	314	99.80	99.71	0.49

**Table 5:** Performance comparison between the proposed method and other state-of-art methods.(*) The results from our implementation and beat-by-beat comparison using AAMI standard [26].

Database	Method	Year	Total Beats	S(%)	P(%)	DER(%)
**MITDB**	This work		109494	99.80	99.71	0.49
	Elgendi’s algorithm (*)	2013	109494	99.89	99.48	0.63
	Elgendi’s algorithm [10]		109985	99.78	99.87	*N*/*A*
	Pan-Tompkins’s algorithm [11]	1986	109494	99.20	99.08	1.72
**NSTDB** [27]	This work		25590	97.12	95.03	7.95
	Elgendi’s algorithm (*)	2013	25590	98.80	89.54	12.73
	Elgendi’s algorithm [10]		26370	95.39	90.25	*N*/*A*
	Pan-Tompkins’s algorithm [11]	1986	25590	99.06	87.95	14.51
**QTDB** [28]	This work		85353	99.94	99.78	0.29
	Elgendi’s algorithm (*)	2013	85353	99.76	99.42	0.82
	Elgendi’s algorithm [10]		111201	99.99	99.67	*N*/*A*
	Pan-Tompkins’s algorithm [11]	1986	85353	99.60	98.35	2.07
**LSTDB** [29]	This work		76181	99.92	99.70	0.98
	Elgendi’s algorithm (*)	2013	76181	99.42	99.70	1.27
	Pan-Tompkins’s algorithm [11]	1986	76181	91.78	98.95	3.3
**TWADB** [30]	This work		19003	99.01	99.14	1.10
	Xiao Hu’s algorithm [41]	2014	79681	100	100	0
	Elgendi’s algorithm (*)	2013	19003	97.21	99.54	1.31
	Elgendi’s algorithm [10]		19003	98.88	99.12	*N*/*A*
	Pan-Tompkins’s algorithm [11]	1986	19003	88.32	94.04	18.23
**SVDB** [31]	This work		182499	99.90	99.8	0.6
	Elgendi’s algorithm (*)	2013	182499	99.85	99.70	0.8
	Elgendi’s algorithm [10]		184744	99.96	99.80	*N*/*A*
	Pan-Tompkins’s algorithm [11]	1986	182499	99.86	99.56	0.57
**NSRDB** [32]	This work		183092	100.00	99.96	0.5
	Elgendi’s algorithm (*)	2013	183092	99.82	99.60	0.7
	Elgendi’s algorithm [10]		183092	99.99	99.96	*N*/*A*
	Pan-Tompkins’s algorithm [11]	1986	183092	99.91	99.97	0.60
**LAFDB** [33]	This work		6705	99.59	96.11	8.5
	Elgendi’s algorithm (*)	2013	6705	99.49	93.11	9.6
	Elgendi’s algorithm [10]		7618	99.59	94.11	*N*/*A*
	Pan-Tompkins’s algorithm [11]	1986	6705	64.21	99.01	14.56

**Table 6:** Comparisons of the DER from the proposed method with other studies for ECG records 121, 200, 202, 217, 105, and 108.

Methods	Year	Record					
		121	202	200	217	105	108
This work		0.16	0.09	0.35	0.09	1.21	1.53
Quadratic filtering [5]	2015	0.00	0.00	0.19	0.27	1.59	4.08
Wavelet transform [25]	2014	0.10	0.09	0.30	0.23	0.81	**8.4**
Elgendi’s algorithm [10]	2013	0.11	0.19	0.23	0.18	**1.87**	1.59
Linear filtering [45]	2013	0.11	0.09	0.15	0.09	1.25	0.57
S-transform [46]	2010	0.16	0.09	0.23	0.23	1.24	2.44
Artificial neural network [39]	2014	0.16	0.33	0.31	**0.64**	0.23	0.51
Mathematical morphology [47]	2009	**0.70**	**0.37**	0.50	0.23	1.01	0.68
Adaptive Mathematical morphology [48]	2016	0.11	0.09	0.19	0.45	1.44	1.13
Four QRS waveform templates [49]	2017	0.11	0.19	**0.73**	0.14	1.61	2.4

**Table 7:** Comparison of processing time for NSRDB of Elgendi’s Algorithm and the proposed work running on MATLAB.

Methods	Error Rate (%)	Processing Time(Second)
Pan-Tompkin’s algorithm	1.72	101.54
Elgendi’s algorithm	0.69	40.99
Proposed algorithm	0.49	7.62
